# Supplementary material for: Tead1a Initiates Transcriptional Priming Through the TEAD1a/YAP‐Notch1‐Spi1/Cebpα Axis to Promote Neutrophil Fate
Source: Adv Sci (Weinh). 2025 Aug 28;12(41):e05441. doi: 10.1002/advs.202505441 (PMC12591127; doi:10.1002/advs.202505441)
Supplement: Supplementary file 4 — Supplementary Dataset [file ADVS-12-e05441-s004.zip › 3-2.zip!/3-2/02-2.cluster/cluster3/Enrich/ego_all.pdf]

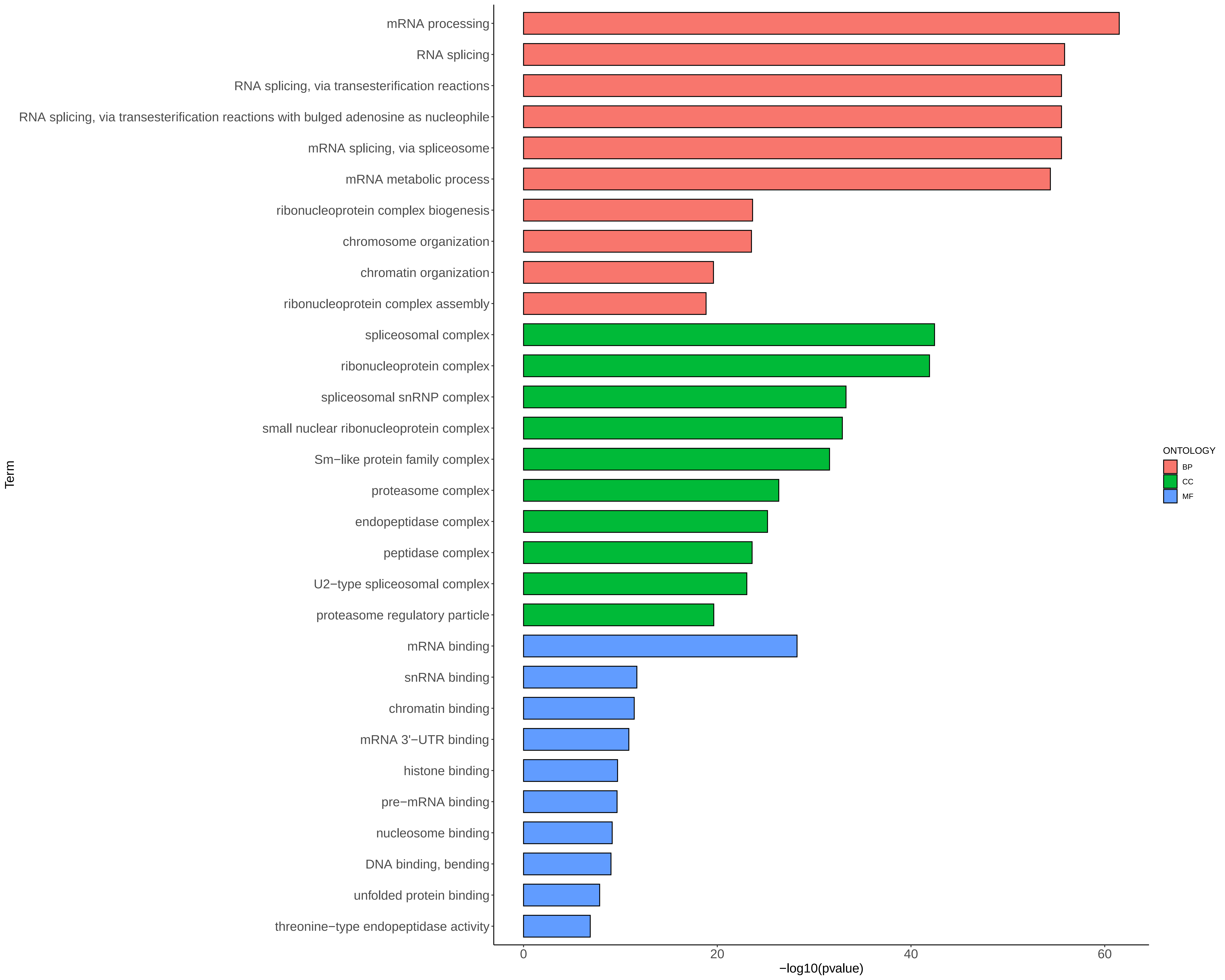

mRNA processing
RNA splicing
RNA splicing, via transesterification reactions
RNA splicing, via transesterification reactions with bulged adenosine as nucleophile
mRNA splicing, via spliceosome
mRNA metabolic process
ribonucleoprotein complex biogenesis
chromosome organization
chromatin organization
ribonucleoprotein complex assembly
spliceosomal complex
ribonucleoprotein complex
spliceosomal snRNP complex
small nuclear ribonucleoprotein complex
Sm−like protein family complex
proteasome complex
endopeptidase complex
peptidase complex
U2−type spliceosomal complex
proteasome regulatory particle
mRNA binding
snRNA binding
chromatin binding
mRNA 3'−UTR binding
histone binding
pre−mRNA binding
nucleosome binding
DNA binding, bending
unfolded protein binding
threonine−type endopeptidase activity
Term
0
20
40
60
−log10(pvalue)
ONTOLOGY
BP
CC
MF
